# Gut microbial metabolites of amino acids in liver diseases

**DOI:** 10.1080/19490976.2025.2586328

**Published:** 2025-11-24

**Authors:** Mengyao Hu, Yi Xu, Hongwei Zhou, Xiaolong He

**Affiliations:** a Microbiome Medicine Center, Department of Laboratory Medicine, Zhujiang Hospital, Southern Medical University, Guangzhou, Guangdong, People's Republic of China; b Guangdong Provincial Key Laboratory of Single-cell and Extracellular Vesicles, Southern Medical University, Guangzhou, People's Republic of China; c Department of Gastroenterology, Shenzhen Hospital, Southern Medical University, Shenzhen, People's Republic of China; d State Key Laboratory of Organ Failure Research, Southern Medical University, Guangzhou, Guangdong, People's Republic of China; e Department of Critical Care Medicine, Zhujiang Hospital, Southern Medical University, Guangzhou, People's Republic of China

**Keywords:** Amino acids metabolism, gut microbiota, microbial metabolites, liver diseases

## Abstract

Due to the unique anatomical connection between the gut and liver, metabolites derived from gut microbiota are closely linked to hepatic health and disease. Among these microbial metabolites, amino acid derivatives have emerged as crucial mediators in gut-liver communication, influencing liver pathophysiology through their bioactive properties. Accumulating evidence demonstrates that amino acid metabolites, including amines, indoles, aromatic derivatives, branched-chain fatty acids (BCFAs), and sulphur-containing compounds (SCCs), function as critical signaling molecules within the gut-liver axis. These metabolites modulate immune responses, inflammation, and metabolic homeostasis, thus affecting the progression and severity of liver diseases. Understanding the specific microbial metabolic pathways responsible for producing these metabolites is essential to reveal novel pathological mechanisms and identify promising biomarkers and therapeutic targets. In this review, we summarize recent advances in microbial amino acid metabolism and highlight the diverse roles of amino acid-derived metabolites in liver disease progression. Furthermore, we discuss the translational potential and current challenges associated with targeting microbial amino acid metabolism in clinical settings. A deeper understanding of these metabolic interactions may ultimately facilitate the development of innovative diagnostic tools and effective therapeutic strategies for liver diseases.

## 1. Introduction

The gut and liver engage in extensive bidirectional communication primarily via the biliary tract, portal vein, and systemic circulation, a dynamic interplay referred to as the gut-liver axis. A central component of gut-liver axis is the gut microbiota, a rich and diverse microbial ecosystem that has co-evolved with the host. In recent years, it has been increasingly recognised as a “metabolic organ” capable of generating a wide range of bioactive molecules.^1^ Among these, short-chain fatty acids (SCFAs) and bile acids have been extensively reviewed for their profound effects on host physiology and pathophysiology. Meanwhile, microbial amino acid metabolites have emerged as a novel and increasingly studied class of signalling molecules.

Proteins that escape digestion in the small intestine reach the colonic lumen, where they undergo microbial proteolysis and are degraded into amino acids. The resulting amino acids are subsequently metabolised through a series of microbial biochemical reactions, including fission, deamination, decarboxylation, oxidation, and reduction, leading to a diverse array of structurally related end products.^2^ These metabolites exhibit considerable chemical and functional diversity, and many have been identified as important mediators of host-microbiota crosstalk within the gut-liver axis. Although previous reviews have discussed specific types of amino acid metabolism by the gut microbiota or its roles in certain diseases, a comprehensive and systematic overview of its relevance to liver health is still lacking.^3-7^ This review aims to integrate current research advances by outlining the microbial catabolic pathways of amino acids and their metabolites, further elucidating the roles of key amino acid metabolites in liver-related diseases, and exploring the translational potential of targeting microbial amino acid metabolism for therapeutic applications. The gut microbiota includes bacteria, fungi, and other microorganisms. Given that fungal amino acid metabolism is less well characterised and its metabolites have rarely been linked to liver diseases, this review focuses on bacterial metabolism.

## 2. Regulation of gut microbiota on amino acid metabolism

Gut microbiota metabolise dietary and endogenous amino acids through enzymatic pathways that are often specific to certain amino acids and enriched in particular microbial taxa.^8^ For example, aromatic amino acid (AAA) aminotransferases and arginine deiminases are predominantly found in *Firmicutes* and *Actinomycetota*, whereas branched-chain amino acid (BCAA) aminotransferases are primarily observed in *Bacteroidota* and *Firmicutes*.^9^ Different bacterial genera have been reported to participate in the metabolism of various amino acid types, including AAAs, BCAAs, sulphur-containing amino acids (SAAs), and basic amino acids (BAAs), producing numerous metabolites that influence host health and disease.^10^

### 2.1. Aromatic amino acids

**Figure 1.**
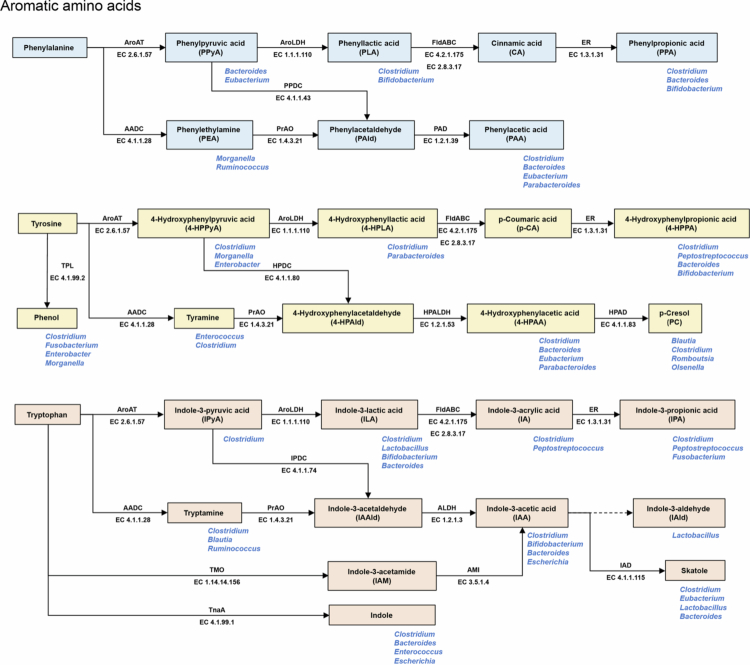
Gut bacterial catabolism of aromatic amino acids. Solid arrows indicate reactions catalysed by established enzymes. Dashed arrows denote steps for which the specific enzymes are unknown. Enzyme names and EC numbers are annotated on solid arrows. Blue labels in the lower-right corner mark representative bacterial genera reported to produce the indicated metabolites. Abbreviations: AADC, aromatic amino acid decarboxylase; ALDH, aldehyde dehydrogenase; AMI, amidase; AroAT, aromatic amino acid transaminase; AroLDH, aromatic lactate dehydrogenase; ER, 2-enoate reductase; FldABC, phenyllactate dehydratase complex; HPAD, 4-hydroxyphenylacetate decarboxylase; HPALDH, 4-hydroxyphenylacetaldehyde dehydrogenase; HPDC, 4-hydroxyphenylpyruvate decarboxylase; IAD, indoleacetate decarboxylase; IPDC, indolepyruvate decarboxylase; PAD, phenylacetaldehyde dehydrogenase; PPDC, phenylpyruvate decarboxylase; PrAO, primary-amine oxidase; TMO, tryptophan 2-monooxygenase; TnaA, tryptophanase; TPL, tyrosine phenol-lyase.

AAAs, including phenylalanine, tyrosine, and tryptophan, are among the most actively metabolised substrates of the gut microbiota and undergo a series of conserved biochemical transformations (Figure 1). Their shared structural feature, consisting of an aromatic ring linked to the universal amino acid backbone, predisposes them to common microbial pathways such as decarboxylation, transamination, and redox reactions. AAA decarboxylases catalyse the conversion of phenylalanine, tyrosine, and tryptophan into phenylethylamine (PEA), tyramine, and tryptamine, respectively. Although these enzymes are widely distributed among gut bacteria, their substrate specificities differ across taxa. Evidence from strain-level *in vitro* studies indicates that *Morganella morganii* predominantly produces PEA, whereas *Clostridium asparagiforme* and *Enterococcus faecalis* favour the formation of tyramine. Tryptamine production is more characteristic of *Clostridium sporogenes*, *Tyzzerella nexilis*, and *Blautia hansenii*. In *Ruminococcus gnavus*, the levels of PEA and tryptamine are considerably higher than that of tyramine.^11-14^ Another shared route of AAA metabolism is transamination catalysed by aromatic amino acid transaminase (AroAT), which generates the keto acid intermediates phenylpyruvic acid, 4-hydroxyphenylpyruvic acid, and indole−3-pyruvic acid (IPyA). These intermediates, together with trace amines derived from decarboxylation, can undergo subsequent oxidative and reductive reactions that yield a wide range of downstream products, including acetic, propionic, and aldehyde derivatives. Several gut commensals such as *Bacteroides thetaiotaomicron, Bacteroides eggerthii, Bacteroides ovatus, Bacteroides fragilis, Parabacteroides distasonis, Eubacterium hallii,* and *Clostridium bartlettii* are capable of fermenting all three AAAs, collectively producing phenylacetic acid (PAA), 4-hydroxyphenylacetic acid (4-HPAA), and indole−3-acetic acid (IAA). Notably, *Clostridium bartlettii* showed 100 times higher IAA production than the other species tested.^15^ In addition, many *Clostridium* species have been reported to synthesise a diverse array of aromatic derivatives, although the specific metabolic end products depended upon the species.^16^ Other taxa also contribute, with certain *Lactobacillus* strains produce phenyllactic acid (PLA), while *Peptostreptococcus* species generate indole−3-propionic acid (IPA), indole−3-acrylic acid (IA), and 4-hydroxyphenylpropionic acid (4-HPPA).^17^^,^^18^

Apart from the shared pathways, several distinct microbial conversions of AAAs have been reported (Figure 1). In tyrosine metabolism, *Clostridium, Fusobacterium, Enterobacter, Morganella, Citrobacter, Klebsiella,* and *Olsenella* have been shown to convert tyrosine to phenol, whereas *Blautia, Clostridium, Romboutsia,* and *Olsenella* can further metabolise 4-HPAA into *p*-cresol.^12^ IAA, recognised as one of the most extensively studied indole derivatives, is generated not only through the IPyA and tryptamine pathways but also via the indole−3-acetamide (IAM) pathway catalysed by tryptophan 2-monooxygenase (TMO). While this route is well established in plant- and soil-associated microorganisms, its presence in gut microbiota has rarely been documented.^19^ IAA can subsequently be converted by *Lactobacillus murinus, Lactobacillus acidophilus,* and *Lactobacillus reuteri* into indole−3-aldehyde (IAld),^20^^,^^21^ or by *Bacteroides, Clostridium,* and *Lactobacillus* into skatole.^15^^,^^22^^,^^23^ Furthermore, multiple Gram-positive and Gram-negative taxa encode TnaA tryptophanase, enabling the production of large quantities of indole, a metabolite that plays a pivotal role in microbial consortia and in human health.^24^^,^^25^ Collectively, these observations indicate that taxonomic differences, including strain-level variation, shape the specific metabolite profiles, and that metabolic exchange among coexisting bacteria further expands the repertoire of aromatic derivatives in the gut.

### 2.2. Branched-chain amino acids

**Figure 2.**
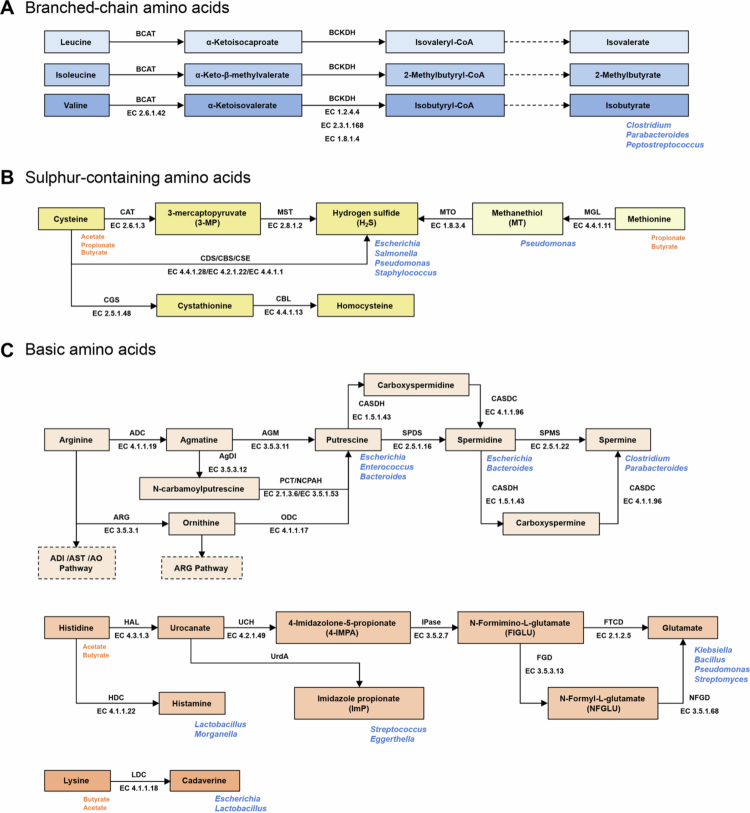
Gut bacterial catabolism of branched-chain, sulphur-containing, and basic amino acids**.** Solid arrows indicate reactions catalysed by established enzymes. Dashed arrows denote steps for which the specific enzymes are unknown. Enzyme names and EC numbers are annotated on solid arrows. Blue labels in the lower-right corner mark representative bacterial genera reported to produce the indicated metabolites. Orange labels in the lower-right list the SCFAs reported to arise from the corresponding amino acid. Dashed box outlines mark pathways that are omitted for brevity. Abbreviations: ADC, arginine decarboxylase; ADI, arginine deiminase; AgDI, agmatine deiminase; AGM, agmatinase; AO, arginine oxidase; ARG, arginase; AST, arginine succinyltransferase; BCAT, branched-chain amino acid transaminase; BCKDH, branched-chain *α*-keto acid dehydrogenase complex; CASDC, carboxyspermidine decarboxylase; CASDH, carboxyspermidine dehydrogenase; CAT, cysteine transaminase; CBL, cystathionine beta-lyase; CBS, cystathionine beta-synthase; CDS, L-cysteine desulfidase; CGS, cystathionine gamma-synthase; CSE, cystathionine *γ*-lyase; FGD, formimidoylglutamate deiminase; FTCD, glutamate formimidoyltransferase; HAL, histidine ammonia-lyase; HDC, histidine decarboxylase; IPase, imidazolonepropionase; LDC, lysine decarboxylase; MGL, methionine gamma-lyase; MST, 3-mercaptopyruvate sulfurtransferase; MTO, methanethiol oxidase; NCPAH, *N*-carbamoylputrescine amidohydrolase; NFGD, *N*-formylglutamate deformylase; ODC, ornithine decarboxylase; PCT, putrescine carbamoyltransferase; SPDS, spermidine synthase; SPMS, spermine synthase; UCH, urocanate hydratase; UrdA, urocanate reductase.

BCAAs, including leucine, isoleucine, and valine, undergo characteristic microbial transformations within the gut in addition to host metabolism (Figure 2A). Their microbial catabolism is initiated by branched-chain amino acid transaminase (BCAT), which converts BCAAs into the corresponding branched-chain *α*-keto acids. These intermediates are then oxidatively decarboxylated by branched-chain keto acid dehydrogenase (BCKDH) to generate the respective acyl-CoA derivatives. These two reactions constitute the common pathway of BCAA degradation, after which the branched-chain metabolites are further processed through three distinct enzymatic systems to ultimately generate BCFAs. Several *Clostridium* species, as well as *Parabacteroides merdae* have been reported to metabolise leucine, isoleucine, and valine into isovalerate, 2-methylbutyrate, and isobutyrate, respectively.^26^^,^^27^ In *Clostridium porogenes* and *Parabacteroides merdae*, this metabolic capacity has been experimentally confirmed to be mediated by the porA gene.^28^^,^^29^

### 2.3. Sulphur-containing amino acids

SAAs, primarily cysteine and methionine, undergo microbial transformations that produce sulphur metabolites (Figure 2B). A well-characterised route is the cleavage of cysteine by cysteine desulfhydrase (CDS), releasing hydrogen sulphide (H₂S). CDS activity has been demonstrated in *Salmonella enterica* serovar Typhimurium and *Escherichia coli*.^30^ It has also been identified in oral microorganisms such as *Fusobacterium nucleatum*, *Prevotella intermedia*, and *Streptococcus anginosus*, where it drives substantial H₂S production that contributes to oral malodor and periodontal disease.^31-33^ Notably, these species are also common commensals of the human colon. In addition to CDS, earlier studies have shown that bacteria can utilise cystathionine *β*-synthase (CBS), cystathionine *γ*-lyase (CSE), and mercaptopyruvate sulfurtransferase (MST) to convert cysteine into H₂S. Functional analyses in pathogens such as *Pseudomonas aeruginosa* and *Staphylococcus aureus* confirmed the involvement of these enzymes in H₂S production,^34^ suggesting that multiple enzymatic strategies for sulphide generation exist in bacteria, although these pathways remain poorly characterised in commensal taxa. Cysteine can also enter the transsulfuration pathway via cystathionine *γ*-synthase (CGS) and cystathionine *β*-lyase (CBL), generating cystathionine and subsequently homocysteine, which functions both as a direct precursor for methionine biosynthesis and as a substrate for the reverse transsulfuration pathway to produce cysteine. Methionine degradation provides another important microbial source of sulphur metabolites. Methionine *γ*-lyase (MGL) catalyses the conversion of methionine into methanethiol, which can be further transformed by methanethiol oxidase (MTO) to yield H₂S. Comparative enzymatic studies have shown that *Pseudomonas ovalis* exhibits the highest MGL activity, and the enzyme is inducibly formed upon addition of methionine to the medium.^35^

### 2.4. Basic amino acids

BAAs, including arginine, histidine and lysine, function as critical metabolic precursors for the intestinal polyamine pool (Figure 2C). The catabolism of arginine in bacteria is mediated through several well-characterised pathways: the arginine deiminase (ADI) pathway, the arginase (ARG) pathway, the arginine succinyltransferase (AST) pathway, the arginine oxidase (AO) pathway, and the arginine decarboxylase (ADC) pathway.^36^^,^^37^ Here we focus on the ADC pathway, which generates agmatine and supports microbial polyamine biosynthesis. In *Enterococcus faecalis*, agmatine deiminase (AgDI) converts agmatine to *N*-carbamoylputrescine, which is then further metabolised into putrescine by putrescine carbamoyltransferase (PCT). This route provides ATP and confers resistance to acid stress.^38^ In *Bacteroides thetaiotaomicron*, *N*-carbamoylputrescine is hydrolysed by *N*-carbamoylputrescine amidohydrolase (NCPAH) to produce putrescine, a distinct step recently validated and kinetically characterised through genetic and biochemical analyses.^39^ In addition to proceeding via agmatine, *Escherichia coli* synthesises putrescine by decarboxylating ornithine, and this putrescine is subsequently converted to spermidine by spermidine synthase (SPDS).^40^ This general mode of spermidine biosynthesis is widespread among bacteria, although pathway specifics differ. In the key gut commensal *Bacteroides thetaiotaomicron*, the carboxyspermidine dehydrogenase/decarboxylase (CASDH/CASDC) system has been shown to be essential for spermidine production.^41^ In comparison, only a few gut commensals produce spermine. *Parabacteroides distasonis*, for example, has been reported to synthesise spermine via spermine synthase (SPMS) and to protect against testicular injury.^42^ Notably, a second spermine pathway involving carboxyspermine has recently been identified in *Clostridium leptum*, underscoring that additional bacterial routes of polyamine biosynthetic pathways likely remain to be discovered.^43^

Histidine is processed by microbes along two principal routes and one emerging side pathway. The first route is decarboxylation to histamine catalysed by histidine decarboxylase (HDC). The probiotic *Lactobacillus reuteri* has repeatedly been shown to convert dietary histidine to histamine which signals through epithelial histamine type−2 receptor (H2R) to suppress colitis and attenuate tumour necrosis factor (TNF), highlighting a distinct immunomodulatory role for histamine.^44^^,^^45^ The second route is the classical histidine utilisation pathway in which histidine is deaminated by histidine ammonia-lyase (HAL) to urocanate, then transformed by urocanase (UCH) to 4-imidazolone−5-propionate, and then converted by imidazolonepropionase (IPase) to *N*-formimino-L-glutamate (FIGLU). Downstream processing of FIGLU varies across taxa. In genera such as *Klebsiella* and *Bacillus*, FIGLU can be hydrolysed directly to L-glutamate, whereas in others such as *Pseudomonas* and *Streptomyces* the formimino group is first removed to generate *N*-formyl-L-glutamate (NFGLU), which is then hydrolysed to formate and L-glutamate that enter glutamate metabolism.^46^ In parallel, a side pathway yielding imidazole propionate (ImP) has gained attention. Certain gut bacteria encode urocanate reductase (UrdA), for which no Enzyme Commission number has yet been assigned, that reduces urocanate to ImP. Levels of ImP are elevated in individuals with type 2 diabetes (T2D), and ImP impairs glucose tolerance and insulin signalling when administered to mice. The species *Streptococcus mutans* and *Eggerthella lenta* have been implicated in this association.^47^ Recent studies further implicate ImP in atherosclerosis, highlighting that many microbially derived amino acid metabolites with important biological functions have yet to be identified.^48^ Beyond these signature products, colonic fermentation of histidine contributes SCFAs including acetate and propionate.

Lysine is also an important substrate in bacterial polyamine metabolism and is the principal precursor of cadaverine. In *Escherichia coli* and *Lactobacillus*, lysine decarboxylase (LDC) converts L-lysine to cadaverine, and lysine/cadaverine antiporter exchange extracellular lysine for intracellular cadaverine to support acid tolerance.^49^^,^^50^ Beyond polyamine formation, lysine also serves as a significant source of microbial butyrate in the gut. The lysine route is one of the four main bacterial pathways for butyrate formation, alongside the acetyl-CoA, 4-aminobutyrate, and glutarate pathways.^51^ In addition, cadaverine undergoes transamination and dehydrogenation to yield 5-aminovalerate (5-AVA), which is subsequently oxidised to glutarate, thereby intersecting with the butyrate biosynthetic pathway.^52^ Aside from butyrate, lysine can also be metabolised to acetate, which modulates the efficiency of butyrate formation.^53^ This capacity for SCFA production has been demonstrated for both BAAs and SAAs in a faecal microbiota fermentation study (Figure 2B, C).^54^

Together, microbial amino acid metabolism is highly complex, involving numerous intermediate products, side reactions, and enzymatic pathways that may vary across taxa. Certain metabolites can be produced through multiple, overlapping routes involving different microbial species or enzyme isoforms. For example, phenylacetic acid can be generated either via the oxidative deamination of PEA, a decarboxylation product of phenylalanine,^55^ or through the conversion of phenylpyruvate, a transamination product of the same amino acid.^56^ Such metabolic flexibility illustrates the redundancy and interconnectedness of microbial pathways. Beyond this complexity, microbial amino acid metabolism also harbours considerable unknowns. Recent studies have revealed that gut bacteria can convert tyrosine into homovanillic acid, a compound previously thought to arise predominantly from host dopamine metabolism, suggesting that many microbial products remain undiscovered.^57^ Continued exploration of these pathways will be essential for fully understanding the contribution of microbial amino acid metabolism to host physiology and disease.

## 3. Microbial amino acid metabolites as signalling molecules in the gut-liver crosstalk

**Figure 3.**
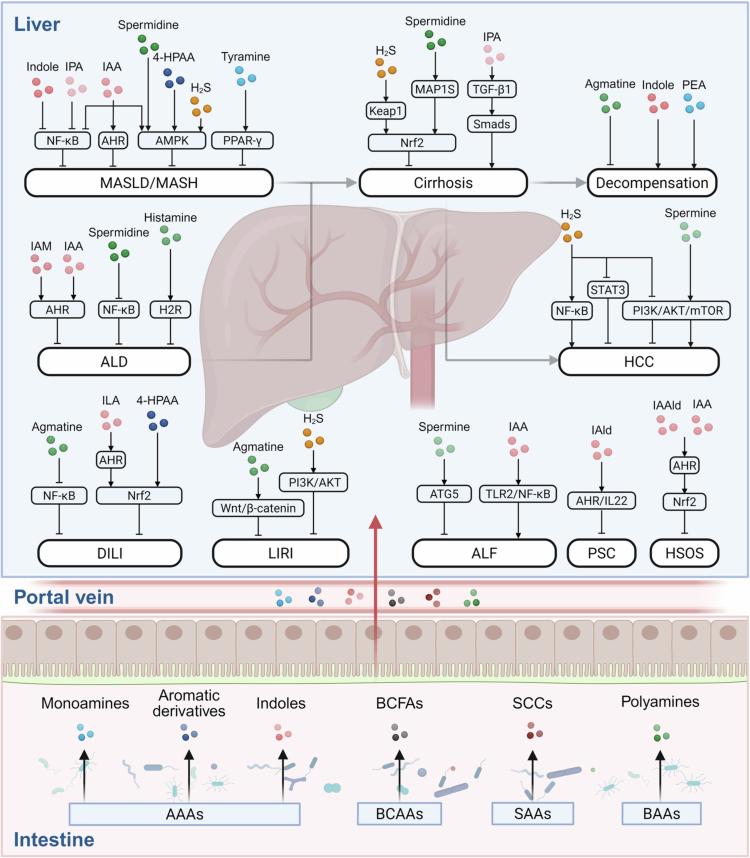
Microbial amino acid metabolism and its representative signalling pathways in the gut-liver axis. Gut microbiota metabolise AAAs, BCAAs, SAAs, and BAAs into a variety of bioactive compounds, including monoamines, indoles, aromatic derivatives, BCFAs, SCCs, and polyamines. These microbial metabolites can translocate to the liver via the portal circulation and contribute to the pathogenesis and progression of liver diseases, including ALD, MASLD, MASH, cirrhosis, HCC, DILI, LIRI, ALF, PSC, and HSOS. Regular arrows indicate gene activation and detrimental effects on disease, while blunt arrows indicate gene inhibition and protective effects on disease. Figure created in BioRender. Abbreviations: 4-HPAA, 4-hydroxyphenylacetic acid; AAAs, aromatic amino acids; AHR, aryl hydrocarbon receptor; AKT, protein kinase-B; ALD, alcohol-associated liver disease; ALF, acute liver failure; AMPK, AMP-activated protein kinase; ATG5, autophagy-related 5; BAAs, basic amino acids; BCAAs, branched-chain amino acids; BCFAs, branched-chain fatty acids; DILI, drug-induced liver injury; H2R, histamine type−2 receptor; H_2_S, hydrogen sulphide; HCC, hepatocellular carcinoma; HSOS, hepatic sinusoidal obstruction syndrome; IAA, indole−3-acetic acid; IAM, indole−3-acetamide; IPA, indole−3-propionic acid; Keap1, kelch-like ECH-associated protein 1; LIRI, liver ischaemia reperfusion injury; MAP1S, microtubule-associated protein 1S; MASH, metabolic dysfunction-associated steatohepatitis; MASLD, metabolic dysfunction-associated steatotic liver disease; mTOR, mammalian target of rapamycin; NF-κB, nuclear factor κB; Nrf2, nuclear factor (erythroid-derived 2)-like 2; PEA, phenylethylamine; PI3K, phosphatidylinositol 3-kinase; PPAR-*γ*, peroxisome proliferator-activated receptor γ PSC, primary sclerosing cholangitis; SAAs, sulphur-containing amino acids; SCCs, sulphur-containing compounds; STAT3, signal transducer and activator of transcription 3; TGF-β1, transforming growth factor-β1; TLR, toll-like receptor.

Growing evidence indicates that microbial amino acid metabolites act as important signalling molecules within the gut-liver axis. These metabolites primarily include amines, indoles, aromatic derivatives, BCFAs, SCCs, and ammonia (Table 1 and 2). Among them, ammonia, a well-recognised microbial byproduct of amino acid deamination, plays a significant role in hepatic encephalopathy (HE) and other liver diseases, and its underlying mechanisms have been extensively reviewed elsewhere.^58^ Thus, in this section, we focus on the roles and mechanisms of the other five classes of metabolites in metabolic disorders such as insulin resistance (IR) and obesity, as well as in liver diseases including metabolic dysfunction-associated steatotic liver disease (MASLD), metabolic dysfunction-associated steatohepatitis (MASH), alcohol-associated liver disease (ALD), acute liver failure (ALF), cirrhosis, and hepatocellular carcinoma (HCC) (Figure 3).

**Table 1.** Human gut microbial amino acid-derived metabolite signatures in liver diseases.

Metabolites	Related liver disease	Study objects	Sample types	Trends in disease	References
**Amines**					
Phenylethylamine (PEA)	HE	Patients with cirrhosis *vs* patients with HE	Serum	Increase	[59]
Tyramine	MASLD	Children with simple obesity *vs* children with obesity accompanied by MASLD	Serum	Increase	[60]
Cadaverine	MASLD	Subjects with normal liver *vs* subjects with MASLD	Faeces	Increase	[61]
Histamine	Biliary atresia	Normal controls *vs* infants with biliary atresia	Liver tissues	Increase	[62]
Putrescine	MASLD	Healthy controls *vs* patients with MASLD	Liver tissues	Increase	[63]
Spermine	HCC	Healthy controls *vs* patients with HCC	Serum	Increase	[64]
**Indoles**					
Indole	MASLD	Lean subjects *vs* overweight subjects *vs* obese subjects	Serum	Decrease	[65]
Indole−3-acetic acid (IAA)	MASLD	Healthy controls *vs* patients with MASLD	Faeces	Decrease	[66]
	ALD	Healthy controls *vs* patients with alcoholic hepatitis	Faeces	Decrease	[67]
Indole−3-acrylic acid (IA)	Obesity	Healthy controls *vs* patients with obesity	Serum	Decrease	[68]
Indole−3-propionic acid (IPA)	Obesity	Lean controls *vs* obese T2D subjects	Plasma	Decrease	[69]
	Obesity	Normal-weight subjects *vs* overweight subjects	Serum, follicular fluid	Decrease	[70]
	Obesity	Average weight subjects *vs* overweight subjects *vs* obese subjects	Serum, colonic mucosa	Decrease	[71]
	MASLD	Healthy controls *vs* patients with MASLD	Faeces	Decrease	[66]
	Liver fibrosis	Bariatric surgery subjects with fibrosis *vs* those without fibrosis	Serum	Decrease	[72]
**Aromatic Derivatives**					
4-Hydroxyphenyllactic acid (4-HPLA)	MASLD	Individuals without MASLD *vs* individuals with MASLD in twins and families (discovery cohort)Biopsy-proven MASLD patients without advanced fibrosis *vs* those with advanced fibrosis (validation cohort)	Serum	Increase	[73]
Phenylacetic acid (PAA)	MASLD	Non-diabetic women with morbid obesity	Plasma	Increase	[74]
Phenyllactic acid (PLA)	HCC	Healthy controls *vs* patients with HCC	Portal vein serum, liver tissues	Increase	[75]
**Branched-chain fatty acids (BCFAs)**					
2-Methylbutyrate	Obesity	Normal-weight subjects *vs* obese subjects	Faeces	Increase	[76]
	MASLD	T2D patients with *vs* without MASLD	Serum	Decrease	[77]
	MASLD	Healthy controls *vs* patients with MASLD	Plasma	Increase	[78]
Isobutyrate	Obesity	Normal-weight controls *vs* women with morbid obesity	Plasma	Decrease	[79]
	Hypercholesterolaemia	Subjects with normocholesterolemia *vs* subjects with hypercholesterolaemia	Faeces	Increase	[80]
	MASLD	T2D patients with *vs* without MASLD	Serum	Decrease	[77]
	MASLD	Healthy controls *vs* patients with MASLD	Faeces	Increase	[81]
	MASLD	Lean healthy controls *vs* patients with lean MASLD	Serum	Increase	[82]
	Obesity	Normal-weight controls *vs* women with morbid obesity	Plasma	Increase	[79]
	Obesity	Normal-weight subjects *vs* obese subjects	Faeces	Increase	[76]
	Hypercholesterolaemia	Subjects with normocholesterolemia *vs* subjects with hypercholesterolaemia	Faeces	Increase	[80]
**Sulphur-containing compounds (SCCs)**					
Methanethiol (MT)	HE	Healthy controls *vs* cirrhotic patients without overt HE *vs* those with overt HE	Serum	Increase	[83]
Methanethiol (MT)	HE	Healthy controls *vs* patients with different grades of HE	Serum	Increase	[84]

Abbreviations: ALD, alcohol-associated liver disease; HCC, hepatocellular carcinoma; HE, hepatic encephalopathy; MASLD, metabolic dysfunction-associated steatotic liver disease; T2D, type 2 diabetes.

**Table 2.** Roles of gut microbial amino acid-derived metabolites in preclinical models of liver diseases.

Metabolites	Related liver disease	Study objects	Effects	Related signalling pathway	References
**Amines**					
Phenylethylamine (PEA)	IR	Monkeys and mice 3T3-L1 adipocytes	Detrimental	TAAR1/ERK	[85]
	HE	CCl_4_-treated mice	Detrimental	/	[59]
Tryptamine	Obesity	HFD-fed mice3T3-L1 adipocytes	Protective	/	[86]
	IR	Monkeys and mice3T3-L1 adipocytes	Detrimental	TAAR1/ERK	[85]
	MASLD	HFD-fed micePA and LPS-treated RAW 264.7 cells	Protective	/	[87]
Tyramine	IR	HFD-fed DrosophilaHFD-fed mice	Protective	TyrR1/Ca²⁺/CRTC/CREB	[88]
	MASLD	HFD-fed miceHepG2 and THLE3 cells	Detrimental	PPAR-γ	[60]
Agmatine	Obesity	Olanzapine-treated rats	Protective	/	[89]
	MASLD	HFD-fed apoE^-/-^ mice	Protective	/	[90]
	HE	BDL-treated rats	Protective	/	[91]
	ALF	D-GalN/LPS-treated mice	Protective	/	[92]
	Cholestatic liver injury	BDL-treated rats	Protective	/	[93]
	LIRI	Liver ischaemia reperfusion injured miceCobalt chloride-treated AML12 cells	Protective	Wnt/β-catenin	[94]
	DILI	Sodium valproate-treated mice	Protective	NF-κB/iNOS	[95]
	Hepatocyte apoptosis	Primary rat hepatocytes	Detrimental	/	[96]
Histamine	MASLD	High-cholesterol diet-fed H2R^-/-^ mice	Protective	H2R	[97]
	MASH	HFHC diet-fed H1R^-/-^ and H2R^-/-^ mice	Protective	H1R/H2R	[98]
	ALD	Alcoholic diet-fed rat	Protective	H2R	[99]
Putrescine	MASLD	HFCF diet-fed micePA-induced HepG2	Detrimental	/	[63]
	ALF	D-GalN-treated ratsCCl_4_-treated ratsCadmium-treated rats	Protective	/	[100-103]
Spermidine	Obesity	HFD-fed miceHIB-1B preadipocytes or C2C12 myotubes	Protective	/	[104]
	Obesity	HFD-fed mice	Protective	/	[105,106]
	Metabolic syndrome	HFD-fed mice3T3-L1 adipocytes	Protective	/	[107]
	MASLD	WD-fed miceFAA-induced AML12 hepatocytes	Protective	DHPS/DOHH/EIF5A	[108]
	MASLD	HFD-fed micePrimary mouse hepatocytes	Protective	AMPK	[109]
	MASLD	WD-fed miceOA and PA-induced HepG2 cells	Protective	THRSP	[110]
	ALD	Alcoholic diet-fed miceLPS-treated mice	Protective	NF-κB	[111]
	Autoimmune Hepatitis	ConA-treated mice	Protective	/	[112]
	Liver fibrosis	CCl4-treated micePrimary mouse HSCs	Protective	MAP1S/NRF2	[113,114]
	Liver fibrosis-HCC	CCl_4_-treated miceDiethylnitrosamine-treated mice	Protective	MAP1S-mediated autophagy	[115]
Spermine	ALF	TAA-treated miceTAA-treated mouse kupffer cells	Protective	ATG5-mediated autophagy	[116]
	HCC	Hepa1−6 xenograft in mice	Detrimental	PI3K/AKT/mTOR/S6K	[64]
**Indoles**					
Indole	Obesity	ob/ob mice	Protective	/	[117]
	MASLD	HFD-fed micePA and LPS-treated primary hepatocytesLPS-treated RAW264.7 cells and BMDM	Protective	PFKFB3	[65]
	MASLD	LPS-injected miceLPS-treated precision-cut liver slices	Protective	NF-κB	[118]
	MASLD	HFD-fed micePA-treated HepG2 cells	Protective	ACE2	[119]
	Portal hypertension	TAA-treated rats	Detrimental	/	[120]
Indole−3-acetaldehyde (IAAld)	HSOS	Monocrotaline-treated rats Rat primary liver sinusoidal endothelial cells	Protective	AHR/Nrf2	[121]
Indole−3-acetamide (IAM)	ALD	Alcoholic diet-fed miceAlcohol-treated HepG2 cells	Protective	AHR	[122]
Indole−3-acetic acid (IAA)	MASLD	HFD-fed mice	Protective	/	[123]
	MASLD	HFD-fed micePA and LPS-treated RAW 264.7 cells FAA and TNF-*α*-treated HepG2 and AML12 cells	Protective	AHR/Fasn/SREBP1c	[87]
	MASLD	WD-fed micePA and LPS-induced RAW 264.7 cells	Protective	AMPK	[124]
	MASLD	WD-fed mice	Protective	NF-κB	[66]
	ALD	Alcoholic diet-fed mice	Protective	AHR/IL22/REG3G	[67]
	ALF	D-GalN/LPS-treated mice	Detrimental	TLR2/NF-κB	[125]
Indole−3-acrylic acid (IA)	Obesity	HFD-fed zebrafishHuman primary visceral preadipocytes	Protective	STAT1/PPARγ	[68]
Indole−3-aldehyde (IAld)	Hypercholesterolaemia	HFD-fed mice	Protective	/	[126]
	MASLD	HFD-fed miceOA-induced HepG2 cells	Protective	AHR	[127]
	MASLD	HFD-fed mice	Protective	AHR	[128]
	PSC	DDC diet-fed mice	Protective	AHR/IL22	[129]
Indole−3-lactic acid (ILA)	MASLD	HFD-fed miceFFA-induced AML12 hepatocytes	Detrimental	/	[130]
	ALF	D-GalN/LPS-treated mice	Detrimental	/	[125]
	DILI	APAP-treated mice	Protective	AHR/Nrf2	[131]
Indole−3-propionic acid (IPA)	Obesity	HFD-fed miceIFN-*γ* and TNF-*α*-treated T84 cells	Protective	/	[69]
	Obesity	HFD-fed miceOrganoidsNIH3T3 cells	Protective	/	[71]
	Obesity	Standard diet-fed rats	Protective	/	[132]
	MASLD	WD-fed mice	Protective	NF-κB	[66]
	MASH	HFD-fed ratsLPS-treated J774A.1 cells	Protective	NF-κB	[133]
	MASLD-HCC	HFHC-fed miceCholesterol-treated HKCI−2 and HKCI−10 cells	Protective	/	[134]
	Liver fibrosis	CCl4-treated mice	Detrimental	TGF-β1/Smads	[135]
	Liver fibrosis	TGF-β1-treated LX−2 cells	Protective	/	[72]
	ALF	D-GalN/LPS-treated mice	Detrimental	/	[125]
skatole	MASLD	PA-induced HepG2, SNU−449, and Huh7 cells	Protective	/	[136]
**Aromatic Derivatives**					
4-Hydroxyphenylacetic acid (4-HPAA)	Obesity	HFD-fed mice	Protective	/	[137]
	Obesity	HFD-fed mice	Protective	SIRT1	[138]
	MASLD	HFD-fed mice	Protective	AMPK	[139]
	ALD	Alcohol diet-fed *Ppara*-null mice	Protective	/	[140]
	DILI	APAP-treated mice	Protective	Nrf2	[141]
4-Hydroxyphenylpropionic acid (4-HPPA)	MASLD	HFD-fed mice	Protective	/	[142]
Cinnamic acid (CA)	MASLD	db/db miceOA-stimulated HepG2 cells	Protective	/	[143]
	ALD	Alcohol-treated mice	Protective	/	[144]
* p*-Cresol (PC)	MASLD	HFD-fed mice	Detrimental	/	[145]
Phenylacetic acid (PAA)	MASLD	Human primary hepatocytesMice	Detrimental	/	[74]
Phenyllactic acid (PLA)	Obesity	HFD-fed with low dose penicillin treated miceOrganoids	Protective	PPAR-γ	[17]
Phenylpropionic acid (PPA)	DILI	APAP- or CCl_4_-treated mice	Protective	/	[146]
**Branched-chain fatty acids (BCFAs)**					
Isobutyrate	Obesity	FAO rat hepatocytes cells	Detrimental	mTORC1/S6K1	[147]
	MASLD	Primary human hepatocyte spheroid	Detrimental	/	[82]
	Liver injury in IBD	DSS-induced colitis liver injury piglets	Protective	TLR4/MyD88/NF-κB	[148]
Isovalerate	Obesity	FAO rat hepatocytes cells	Detrimental	mTORC1/S6K1	[147]
	Liver fibrosis	CCl_4_-treated miceTNFα-stimulated HepG2 cells	Detrimental	/	[149]
**Sulphur-containing compounds (SCCs)**					
Hydrogen sulphide (H_2_S)	Hepatitis	ConA-induced hepatitis mice	Protective	/	[150]
	MASLD	HFD-fed mice	Protective	/	[151]
	MASLD	HFD-fed miceFat-emulsion-treated L02 and HepG2 cells	Protective	AMPK/mTOR	[152]
	MASH	MCD diet-fed rats	Protective	/	[153]
	Liver fibrosis	CCl_4_-treated rats	Protective	/	[154]
	Liver fibrosis	CCl_4_-treated mice	Protective	/	[155]
	Liver fibrosis	Streptozotocin + HFD-treated LDLr^-/-^ miceCCl₄-treated mice	Protective	Keap1/Nrf2/Lrp1	[156]
	Liver fibrosis	Primary rat HSCsBDL-treated rats	Detrimental	/	[157]
	LIRI	Anoxia/reoxygenation injured primary mouse hepatocytesLiver ischaemia/reperfusion injured mice	Protective	PI3K/AKT1	[158]
	Portal hypertension	TAA-treated rats	Detrimental	/	[159]
	Portal hypertension	BDL-treated rats	Protective	/	[160]
	HCC	L02, SMMC−7721 and Huh−7 cellsSMMC−7721 and Huh−7 xenograft tumours in nude mice	Detrimental (low-dose)Protective (high-dose)	EGFR/ERK/MMP-2PTEN/PI3K/AKT	[161]
	HCC	PLC/PRF/5 and L02 cells	Detrimental	NF-κBSTAT3/COX−2	[162]
	HCC	TNFα-stimulated HEK293 cells	Detrimental	NF-κB	[163]
	HCC	HepG2 and HLE cells	Protective	PI3K/AKT/mTOR	[164]
	HCC	HepG2 and Bel7402 cellsHepG2 xenograft in mice	Protective	STAT3	[165]

Abbreviations: ACE2, angiotensin-converting enzyme 2; AHR, aryl hydrocarbon receptor; AKT, protein kinase-B; ALD, alcohol-associated liver disease; ALF, acute liver failure; AMPK, AMP-activated protein kinase; APAP, acetaminophen; apoE, apolipoprotein E; BDL, bile duct ligation; COX-2, cyclooxygenase-2; CREB, cAMP response element-binding protein; CRTC, CREB-regulated transcription coactivator; DCC, 3,5-diethoxycarbonyl-1,4-dihydrocollidine; D-GalN, D-galactosamine; DHPS, deoxyhypusine synthase; DILI, drug-induced liver injury; DOHH, deoxyhypusine hydroxylase; DSS, dextran sulphate sodium; EGFR, epidermal growth factor receptor; EIF5A, eukaryotic initiation factor 5A; ERK, extracellular signal-regulated kinase; FAA, fatty acid; Fasn, fatty acid synthase; H1R, histamine type-1 receptor; H2R, histamine type-2 receptor; HCC, hepatocellular carcinoma; HE, hepatic encephalopathy; HFCF, high-fat, cholesterol, and fructose; HFD, high-fat diet; HFHC, high-fat/high-cholesterol; HSC, hepatic stellate cell; HSOS, hepatic sinusoidal obstruction syndrome; IBD, inflammatory bowel disease; iNOS, inducible nitric oxide synthetase; IR, insulin resistance; Keap1, kelch-like ECN-associated protein 1; LIRI, liver ischaemia reperfusion injury; LPS, lipopolysaccharides; Lrp1, low-density lipoprotein receptor-related protein 1; MAP1S, microtubule associated protein 1S; MASH, metabolic dysfunction-associated steatohepatitis; MASLD, metabolic dysfunction-associated steatotic liver disease; MCD, methionine- and choline-deficient; MMP-2, matrix metalloproteinase 2; mTOR, mammalian target of rapamycin; MyD88, myeloid differentiation primary response 88; NF-κB, nuclear factor-κB; Nrf2, nuclear factor (erythroid-derived 2)-like 2; OA, oleic acid; PA, palmitic acid; PFKFB3, 6-phosphofructo-2-kinase/fructose-2,6-biphosphatase 3; PI3K, phosphatidylinositol 3-kinase; PPAR-γ, peroxisome proliferator-activated receptor γ PSC, primary sclerosing cholangitis; PtdIns3K, phosphatidylinositol 3-kinase; PTEN, phosphatase and tensin homologue deleted on chromosome ten; REG3G, regenerating islet-derived 3 gamma; S6K, ribosomal protein S6 kinase; SIRT1, sirtuin 1; SREBP1c, sterol regulatory element-binding protein-1c; STAT, signal transducer and activator of transcription; T2D, type 2 diabetes; TAA, thioacetamide; TAAR1, trace amine-associated receptor 1; TGF-β1, transforming growth factor-β1; THRSP, thyroid hormone-responsive protein; TLR, toll-like receptor; TyrR1, tyramine receptor 1; WD, western diet.

### 3.1. Amines

Amines represent a chemically diverse group of nitrogen-containing compounds that are primarily produced in the gut through microbial decarboxylation of amino acids. This group includes monoamines such as PEA, tyramine, and tryptamine, which are derived respectively from phenylalanine, tyrosine, and tryptophan through direct decarboxylation. Polyamines such as cadaverine, agmatine, putrescine, spermidine, spermine, and histamine originate from amino acid precursors including lysine, arginine, ornithine, and histidine, often involving multistep biosynthetic pathways. While certain polyamines can also be synthesised by host cells, the gut microbiota, particularly in the colon, is considered a major source under physiological conditions. In healthy adults, colonic luminal concentrations of polyamines range from the micro- to millimolar level, with putrescine being the most abundant, followed sequentially by spermine, spermidine, and cadaverine.^166^^,^^167^

PEA, tyramine, and tryptamine are classified as trace amines capable of influencing neurotransmission even at low tissue concentrations. Under physiological conditions, they are rapidly degraded by liver monoamine oxidases, thereby limiting their systemic accumulation and neuroactive effects.^55^ However, our recent study suggests that under cirrhotic conditions, reduced monoamine oxidase-B activity in the liver and circulation impairs the clearance of PEA, primarily produced by *Ruminococcus gnavus*, leading to its accumulation in the brain and contributing to the neurological symptoms characteristic of HE.^59^ Beyond their classical roles in the nervous system, these microbial amines have recently gained attention for their emerging involvement in metabolic disorders. Among them, tyramine has been found to be elevated in children with MASLD and positively associated with clinical indicators of hepatic steatosis. Experimental studies further demonstrate that *Enterococcus faecium* B6-derived tyramine promotes MASLD progression via activation of peroxisome proliferator-activated receptor *γ* (PPAR-*γ*) signalling.^60^ Interestingly, in parallel studies using high-fat diet (HFD) models in both Drosophila and mice, tyramine was shown to alleviate IR, which is considered an early initiating factor in the development of MASLD.^88^ Tryptamine, another trace amine, is reported to be reduced in HFD-fed mice, and in vitro studies suggest that tryptamine supplementation attenuates macrophage inflammation, indicating a potential protective role in fatty liver disease.^87^ Similarly, in HFD models, tryptamine improves obesity and IR by directly modulating lipogenesis and lipolysis in white adipose tissue.^86^ However, under physiological conditions, tryptamine has been reported to impair insulin signalling through activation of the trace amine-associated receptor 1 (TAAR1)-extracellular signal-regulated kinase (ERK) signalling axis, suggesting that its metabolic effects may be context dependent.^85^ Given the multifaceted roles of trace amines in metabolic regulation, further research is warranted to elucidate the underlying mechanisms and assess the clinical relevance of trace amines in MASLD.

Histamine, a biogenic amine derived from the decarboxylation of histidine, has been implicated in a range of liver diseases. Clinical studies have reported elevated histamine levels in patients with advanced liver diseases. In individuals with advanced chronic liver disease, high plasma histamine is associated with circulatory dysfunction and independently associated with increased risks of acute-on-chronic liver failure and liver-related mortality.^168^ Similarly, hepatic histamine levels are markedly increased in infants with biliary atresia and correlate positively with the severity of liver fibrosis, suggesting a potential link between histamine accumulation and disease progression.^62^ In contrast, experimental animal models suggest a protective role for histamine in the early stages of liver injury. In alcohol-fed rats, histamine confers protection against hepatic damage via H2R signalling.^99^ Likewise, in dietary models of MASLD and MASH, histamine signalling through histamine type−1 receptor (H1R) and H2R has been shown to regulate glucose and lipid metabolism and attenuate steatohepatitis progression.^97^^,^^98^ These findings suggest stage-specific roles for histamine in liver disease. While elevated levels in patients may result from reduced hepatic clearance, receptor-dependent effects observed in animal models point to potential metabolic benefits. However, the pathological significance of histamine accumulation in advanced liver disease remains unclear and requires further elucidation.

Cadaverine, a polyamine derived from lysine decarboxylation, has been less extensively studied within the gut-liver axis. Limited clinical evidence indicates that faecal cadaverine levels are elevated in patients with MASLD compared to obese individuals with normal liver histology, suggesting a possible association with disease progression.^61^ Further studies are needed to clarify its mechanistic role and clinical significance in liver diseases.

Another subset of polyamines is derived from microbial arginine metabolism. Among them, agmatine is generated through the decarboxylation of arginine and acts as both a precursor and a regulator of downstream polyamines, including putrescine, spermidine, and spermine. These metabolites are involved in diverse physiological and pathological pathways. Notably, agmatine and spermidine have been extensively investigated in experimental models of liver injury. In models of D-galactosamine and lipopolysaccharide (D-GalN/LPS)-induced liver failure, bile duct ligation-induced cholestasis, ischaemia-reperfusion injury, and sodium valproate-induced hepatotoxicity, agmatine administration mitigates hepatic injury by exerting anti-inflammatory, antioxidant, and cytoprotective effects.^92-95^ Additionally, in HFD-fed apolipoprotein E-knockout mice, agmatine reduces hepatic steatosis, suggesting a beneficial role in metabolic liver disease.^90^ Similarly, spermidine has demonstrated beneficial effects in obesity, MASLD, and metabolic syndrome. It improves hepatic lipid metabolism, reduces steatosis, and alleviates systemic metabolic dysfunction through multiple mechanisms, including AMP-activated protein kinase (AMPK) activation, enhancement of mitochondrial function, and gut microbiota-mediated modulation of bile acid metabolism.^104-110^ Additionally, spermidine has been shown to protects against alcohol- and LPS-induced liver injury by reducing oxidative stress and inflammation,^111^ mitigates hepatic fibrosis through Nuclear factor (erythroid-derived 2)-like 2 (Nrf2)-dependent pathways,^113^ and prevents fibrosis-associated hepatocellular carcinoma via MAP1S (Microtubule-associated protein 1S)-mediated autophagy.^115^ Collectively, these findings underscore the broad hepatoprotective properties of agmatine and spermidine and support their therapeutic potential in liver disease management. Further human studies are warranted to evaluate their clinical relevance. Unlike the broadly protective effects observed with agmatine and spermidine, putrescine and spermine exhibit more variable roles across different liver disease contexts. Putrescine has been reported to alleviate hepatic injury in several acute liver damage models.^100-103^ However, its levels are elevated in hepatic tissue of both diet-induced MASH mice and patients with MASH, and in vitro studies suggest that putrescine may exacerbate palmitic acid-induced lipotoxicity in hepatocytes.^63^ Spermine similarly exerts protective effects in thioacetamide-induced acute liver injury by attenuating inflammatory responses in liver-resident macrophages through ATG5-dependent autophagy.^116^ In contrast, increased spermine levels have been detected in the serum of patients with HCC compared to healthy individuals, and mechanistic studies indicate that spermine facilitates M2 macrophage polarisation, thereby impairing antitumor immunity.^64^ These findings indicate that while putrescine and spermine may confer protection in acute liver injury, their accumulation in chronic liver disease may contribute to pathogenesis. Further studies are warranted to delineate their mechanistic roles and to evaluate their potential as biomarkers or therapeutic targets.

### 3.2. Indoles

Tryptophan, an essential aromatic amino acid, is metabolised through three major pathways in the gastrointestinal tract: the kynurenine pathway mediated by indoleamine 2,3-dioxygenase 1, the serotonin biosynthesis pathway via tryptophan hydroxylase 1, and the direct transformation by the gut microbiota into various bioactive molecules.^3^ Metabolites directly derived from microbial tryptophan metabolism, including indole and its derivatives, have emerged as important regulators of host physiology and disease.^169^ Several bacterial genera, including *Clostridium*, *Lactobacillus*, and *Blautia*, have been reported to produce high levels of indole-related substances.^170^ Multiple studies have quantified the levels of indole derivatives in the faeces of healthy adults.^171-174^ However, reported concentrations vary considerably due to differences in analytical methods and interindividual variability. Despite these discrepancies, indole is generally recognised as the most abundant microbial tryptophan catabolite in adults, followed by IAA and IPA. In contrast, the concentrations of several other tryptophan-derived metabolites, such as indole-lactic acid (ILA), IA, IAM, IAld, and indole−3-acetaldehyde (IAAld), have not yet been comprehensively assessed in the human gut.

Indole and several of its derivatives, including ILA, IA, IPA, IAAld, IAld, and IAA are considered endogenous ligands of the aryl hydrocarbon receptor (AHR).^21^^,^^175-177^ AHR signalling is a critical regulator of intestinal and hepatic immune responses, playing an important role in intestinal homoeostasis and liver diseases.^178-181^ A recent study demonstrated that metabolic syndrome is linked to a reduced microbial capacity to produce AHR-activating tryptophan metabolites, and restoring this function through AHR agonists or a *Lactobacillus* strain with high ligand-producing capacity improves glucose metabolism and liver steatosis by enhancing intestinal barrier function and stimulating GLP−1 secretion.^182^ Similar to AHR, pregnane X receptor (PXR) is capable of sensing certain microbial tryptophan catabolites. Among them, IPA has been identified as a ligand for PXR, particularly in the presence of indole, contributing to the regulation of intestinal barrier function.^183^ Importantly, PXR has been recognised as a key regulator in metabolic disorders and chronic liver diseases.^184^^,^^185^ These findings underscore the critical role of bacterial tryptophan catabolites in modulating host metabolic homoeostasis and highlight their potential as therapeutic targets in metabolic disorders and liver diseases. Indole has also been identified as a microbial signalling molecule capable of stimulating the secretion of glucagon-like peptide−1 (GLP−1) from colonic enteroendocrine L cells, a hormone involved in regulating insulin secretion, appetite, and energy balance.^186^ In both animal models and in vitro studies, indole has been shown to attenuate hepatic steatosis, suppress inflammatory responses, and modulate cholesterol metabolism.^65^^,^^117-119^ Additionally, circulating indole levels are reversely associated with body mass index in human subjects, further supporting its potential role in metabolic regulation.^65^

ILA has been shown to alleviate drug-induced liver injury by activating hepatic AHR/Nrf2 signalling pathway.^131^ In contrast, ILA exacerbates hepatic steatosis in the in vitro and in vivo model of MASLD, suggesting a potential pathogenic role under certain metabolic conditions.^130^ Previous studies have indicated that microbiota-regulated AHR signalling exhibits considerable complexity across different liver diseases, and further research is needed to elucidate the mechanisms underlying these diverse effects.^187^ Reduced levels of IPA have been consistently observed in overweight and obese individuals, indicating its potential protective role in metabolic health. Supporting evidence from both animal and in vitro studies shows that IPA supplementation mitigates weight gain, improves intestinal barrier function, and attenuates obesity-related metabolic disorders.^69-71^^,^^132^ In addition to its role in obesity, IPA has demonstrated beneficial effects in MASLD. A recent study showed that IPA levels are decreased in the stool of patients with MASLD. In a mouse model of Western diet-induced MASLD, administration of IPA effectively alleviated hepatic steatosis and inflammation by suppressing the nuclear factor κB (NF-κB) signalling pathway through a reduction in endotoxin levels and inactivation of macrophages.^66^ Consistently, another study reported that IPA supplementation attenuated MASH progression in rats by enhancing gut barrier function and reducing both systemic and hepatic inflammatory responses.^133^ Notably, in a cholesterol-driven MASLD-HCC model, a high-fat/high-cholesterol diet induced gut microbial dysbiosis and disrupted microbial tryptophan metabolism, resulting in significantly reduced serum levels of IPA compared to a high-fat/low-cholesterol diet. Functional assays further demonstrated that IPA inhibited cholesterol-induced lipid accumulation and hepatocyte proliferation, suggesting a protective role microbiota-derived IPA depletion in the pathogenesis of cholesterol-driven MASLD-HCC. Despite its beneficial effects in metabolic liver diseases, the role of IPA in hepatic fibrosis has yielded conflicting results. In a murine model, IPA was shown to exacerbate carbon tetrachloride (CCl_4_)-induced liver injury and fibrosis by promoting hepatic stellate cell (HSC) activation via the transforming growth factor-β1 (TGF-β1)/Smads pathway.^135^ Conversely, clinical data revealed significantly reduced circulating IPA levels in individuals with liver fibrosis, and in vitro studies demonstrated that IPA inhibited TGF-β1-induced adhesion and migration of LX−2 cells, suggesting a potential antifibrotic effect.^72^ These inconsistent findings may be attributed to differences in experimental models and study designs, highlighting the need for further investigation to clarify the role of IPA in hepatic fibrogenesis.

As an endogenous ligand of the AHR, IAld has demonstrated hepatoprotective effects through AHR-mediated signalling in several studies. IAld, produced by *Lactobacillus acidophilus* KLDS1.0901, was shown to alleviate HFD-induced MASLD in mice, accompanied by reduced hepatic lipid accumulation and increased glycogen content in oleic acid-treated hepatocytes.^127^ Moreover, IAld was found to restore HFD-induced intestinal barrier dysfunction by promoting AHR-mediated intestinal stem cell proliferation, thereby reducing systemic inflammation.^128^ In a murine model of primary sclerosing cholangitis (PSC), localised delivery of IAld to the gut attenuated hepatic inflammation and fibrosis by modulating the gut microbiota and activating the AHR-IL−22 signalling axis, underscoring its therapeutic potential in gut-liver axis-related liver diseases.^129^ Another indole derivative, IAA, has demonstrated consistent hepatoprotective effects in liver diseases. Faecal levels of IAA were significantly reduced in patients with MASLD compared to healthy controls, and IAA supplementation effectively alleviated hepatic steatosis and inflammation in an animal model of MASLD.^66^ Several studies have further shown that IAA reduces hepatic lipid accumulation, oxidative stress, and inflammatory responses through mechanisms involving activation of the AHR and AMPK, underscoring its potential as a multifactorial regulator of hepatic metabolic homoeostasis.^87^^,^^123^^,^^124^ Moreover, faecal levels of IAA are significantly reduced in both patients with alcoholic hepatitis and mice fed a chronic-binge ethanol diet, leading to impaired AHR activation and downregulation of IL−22 and REG3G expression in the gut. Supplementation with IAA restores this signalling pathway, strengthens intestinal barrier function, prevents bacterial translocation, and attenuates ethanol-induced liver injury.^67^

### 3.3. Aromatic derivatives

Aromatic derivatives are gut microbial metabolites primarily produced through the fermentation of aromatic amino acids, such as phenylalanine and tyrosine, via transamination followed by a series of oxidative and reductive reactions.^56^ This class includes compounds such as PAA, PLA, phenylpropionic acid (PPA), cinnamic acid (CA), 4-HPAA, 4-HPPA, 4-hydroxyphenylactic acid (4-HPLA), *p*-cresol (PC), phenol, and others. Many of these compounds are classified as phenolic acids, which have traditionally been regarded as plant-derived secondary metabolites. However, accumulating evidence indicates that microbial fermentation of amino acids also constitutes a significant source.^15^ These aromatic metabolites, despite their varying concentrations, have been extensively investigated for their regulatory roles in the pathogenesis and progression of liver diseases.^188^

Among these, 4-HPAA generally exerts a protective role across various forms of liver disease. In HFD-induced mouse models, 4-HPAA and its analogues have been shown to mitigate obesity, hepatic steatosis, and glucose intolerance. Mechanistically, 4-HPAA exerts its beneficial effects by activating hepatic AMPK signalling, modulating intestinal immune responses, and reducing lipid uptake in the gut mucosa. It also enhances Sirtuin 1 (SIRT1) signalling and induces beige adipogenesis and thermogenesis-related gene expression in white adipose tissue, contributing to improved energy metabolism.^137-139^ Beyond metabolic regulation, 4-HPAA has demonstrated hepatoprotective effects in both alcoholic and drug-induced liver injury models. In Acetaminophen (APAP)-treated mice, 4-HPAA promotes nuclear translocation of Nrf2 and enhances phase II detoxification and antioxidant enzyme activity, thereby attenuating hepatocellular injury.^141^ Similarly, in alcohol-fed peroxisome proliferator-activated receptor alpha knockout (*Ppara*-null) mice, 4-HPAA ameliorates liver damage by reducing inflammation and modulating hepatic lipid metabolism.^140^ Collectively, these findings suggest that 4-HPAA may serve as a multifunctional microbial metabolite with therapeutic potential across a range of liver and metabolic disorders.

Another extensively studied metabolite is PLA, which exhibits context-dependent effects depending on disease stage and host status. In early metabolic disorders such as obesity and MASLD, PLA appears to play a protective role. Clinical observations have shown that circulating PLA levels are negatively correlated with hepatic steatosis,^189^ and in experimental models, *Lactobacillus*-derived PLA was found to upregulate intestinal PPAR-*γ* expression and mitigate metabolic dysfunction caused by early-life antibiotic exposure and HFD feeding.^17^ These findings suggest that PLA may support gut epithelial homoeostasis and metabolic regulation, thereby indirectly benefiting liver health. However, in more advanced liver disease states, PLA may shift from a protective to a detrimental marker. In patients with HCC, PLA was significantly elevated in portal vein and liver tissues, correlating with impaired hepatic function and poor prognosis, raising the possibility that PLA accumulation reflects or contributes to disease progression.^75^ Additionally, in alcohol-related liver injury, PLA has been identified as a conserved urinary biomarker in *Ppara*-null mice across different genetic backgrounds, highlighting its potential value for disease monitoring.^190^

In addition, PAA has emerged as a microbial metabolite of interest due to robust evidence from human studies linking it to liver disease pathogenesis. In a multi-omics analysis of obese women with varying degrees of hepatic steatosis, higher levels of microbial gene pathways for PAA production were observed in patients with steatosis, and circulating PAA levels were significantly associated with hepatic triglyceride accumulation. Functional studies further confirmed that chronic PAA administration and faecal microbiota transplantation from steatotic individuals induced hepatic lipid accumulation and disrupted branched-chain amino acid metabolism in mice, supporting a causative role of PAA in MASLD progression.^74^ In contrast, a Mendelian randomisation study suggested that PAA might exert a protective effect against ALD.^189^ This discrepancy may be attributed to the fact that ALD pathogenesis involves alcohol-induced oxidative stress and immune activation rather than lipid metabolism. Further mechanistic and longitudinal studies are needed to clarify the role of PAA in distinct forms of liver disease and its potential as a therapeutic target.

Several less-characterised aromatic microbial metabolites have also been implicated in liver health. PPA, 4-HPPA, and CA have consistently shown hepatoprotective effects in preclinical models. These compounds have been reported to alleviate hepatic steatosis, oxidative stress, or inflammation in different experimental settings, including high-fat diet feeding, alcohol exposure, and chemically induced liver injury.^142-144^^,^^146^ In contrast, PC was shown to promote steatosis and hepatocellular damage. Notably, 4-HPLA, identified through twin-family genetic modelling, showed a significant genetic correlation with hepatic steatosis and fibrosis, and was linked to specific gut microbial taxa, suggesting a host-microbe co-regulatory mechanism in disease susceptibility.^73^

### 3.4. Branched-chain fatty acids

BCFAs, including isovalerate, 2-methylbutyrate, and isobutyrate, are a type of SCFAs characterised by their unique branched molecular structure. Unlike straight-chain SCFAs, such as acetate, propionate, and butyrate, which are primarily produced through carbohydrates catabolism of intestinal microbes, BCFAs originate from the bacterial catabolism of branched-chain amino acids.^191^ In the human gut, BCFAs represent a minor fraction of total SCFAs, with colonic concentrations averaging 4.6 mmol/kg in the proximal colon and 6.3 mmol/kg in the distal colon, which correspond to approximately 3.4% and 7.5% of total SCFAs, respectively.^192^ While straight-chain SCFAs are widely acknowledged as crucial mediators of the gut-X axis and protect against multiple organ injuries,^193^ the physiological functions of BCFAs remain comparatively underexplored. Therefore, this section focuses on current insights into the roles of BCFAs in the gut-liver axis.

In patients with MASLD, circulating levels of BCFAs have shown inconsistent trends across studies. In a cohort of T2D patients with MASLD, serum concentrations of 2-methylbutyrate and isobutyrate were significantly reduced and negatively correlated with disease severity, suggesting a potential protective role.^77^ However, conflicting findings have been reported in other studies. For instance, one investigation found that serum isobutyrate levels were elevated in lean individuals with MASLD,^82^ while another reported increased plasma levels of 2-methylbutyrate in patients with MASLD, which were positively associated with the degree of hepatic fibrosis.^78^ These discrepancies may reflect differences in host metabolic phenotypes, including obesity status, along with variation in gut microbiota composition and dietary protein intake, all of which may influence BCFA production. Given that obesity, hypercholesterolaemia, and other metabolic disorders are recognised as major risk factors for the development and progression of MASLD, it is important to consider their association with BCFA levels. Elevated faecal concentrations of isovalerate and isobutyrate have been reported in patients with hypercholesterolaemia.^80^ Similarly, in a study of individuals with obesity, faecal levels of both 2-methylbutyrate and isovalerate were found to be elevated compared to normal-weight controls.^76^ In parallel, a female obesity cohort showed that plasma isovalerate levels were significantly increased, whereas isobutyrate levels were decreased in women with morbid obesity. However, this pattern did not exhibit a progressive trend along the histological spectrum from simple steatosis to steatohepatitis, suggesting that these changes may reflect systemic metabolic alterations associated with obesity rather than liver-specific pathology.^79^ Mechanistically, both isobutyrate and isovalerate have been shown to stimulate hepatic glucose production in hepatocyte models, with isovalerate demonstrating a greater capacity to overcome insulin-mediated suppression. These effects are mediated through activation of the mammalian target of rapamycin complex 1/S6 kinase 1 (mTORC1/S6K1) signalling pathway,^147^ which plays a central role in hepatic regulation of ketogenesis and lipogenesis.^194^ In addition, isobutyrate has been reported to induce lipid accumulation in human 3D liver spheroids, potentially via modulation of lipogenesis-related genes, fatty acid metabolism, and bile acid signalling.^82^

Beyond metabolic liver diseases, BCFAs have also been implicated in other forms of liver injury. For instance, isovalerate has been shown to exacerbate tumour necrosis factor alpha (TNF-*α*)-induced apoptosis in HepG2 cells, suggesting a detrimental role in liver damage.^149^ In contrast, in a dextran sodium sulphate (DSS)-induced colitis model in piglets, preventive supplementation with sodium isobutyrate alleviated colitis-associated liver injury, highlighting its hepatoprotective role. This protective effect was associated with the suppression of the toll-like receptor 4 (TLR4)/myeloid differentiation primary response 88 (MyD88)/NF-κB signalling pathway, resulting in reduced expression of pro-inflammatory cytokines including TNF-*α*, IL-1β, and IL−6.^148^ This pathway has been widely implicated in diverse inflammatory processes and thought to mediate the anti-inflammatory effects of butyrate in both the gut and the brain.^195-197^ In addition, isobutyrate helped preserve mitochondrial function, thereby limiting oxidative stress and hepatocyte apoptosis.^148^ Similar effects have also been reported for other SCFAs, including acetate and butyrate.^198^ While this suggests a shared mechanism of action among SCFAs, it remains unclear whether BCFAs can confer comparable protective benefits at lower effective doses, warranting further investigation.

### 3.5. Sulphur-containing compounds

Amino acid-derived SCCs are primarily generated through desulfurization reactions of cysteine and methionine, which produce H_2_S and methanethiol (MT). H_2_S in the colon is produced by both gut microbiota and host cells. Its intracellular concentration is determined by multiple factors, including exogenous H_2_S diffusion, endogenous synthesis from cysteine within the cells, and the cellular capacity for H_2_S oxidation.^199^ Studies have reported that the concentration of sulphide in the colonic contents and faeces of mammals ranges from approximately 0.17 to 3.38 mmol/kg.^200^ This broad variability underscores the complexity of H_2_S homoeostasis and suggests that luminal levels are influenced by a multifactorial interplay involving the host metabolic state, dietary composition, and microbial community structure and activity.

Emerging evidence has highlighted the protective roles of H_2_S in several liver diseases. In drug-induced hepatitis mouse models, gut microbiota-derived H_2_S has been shown to exert hepatoprotective effects by mitigating oxidative stress-mediated liver injury.^150^ In the context of MASLD, exogenous H_2_S supplementation significantly improves hepatic lipid metabolism and enhances antioxidant capacity in HFD-fed mice.^151^ Furthermore, in models of MASH, H_2_S prevents disease progression induced by MCD-diets in rats, possibly through abating oxidative stress and suppressing inflammation.^153^ Mechanistically, it has been implicated in the activation of hepatic autophagy via the AMPK-mTOR signalling pathway, contributing to the reduction of serum triglyceride levels and subsequent improvement of steatotic liver phenotypes.^152^ Consistent with these findings, exogenous H_2_S attenuates CCl_4_-induced hepatotoxicity, liver cirrhosis and portal hypertension by its multiple functions including anti-oxidation, anti-inflammation, cyto-protection and anti-fibrosis.^154^ Cystathionine *γ*-lyase gene knockout mice showed exacerbated liver injury and fibrosis, while administration of H_2_S donors reversed these changes.^155^ Mechanistically, H_2_S exerts its protective effects by inducing Keap1 (Kelch-like ECH-associated protein 1) Cys151-dependent S-sulfhydration, which activates the Nrf2 antioxidant pathway, promotes Nrf2 nuclear translocation, and upregulates antioxidant gene expression, thereby mitigating hepatocyte damage and fibrotic progression.^156^ These observations suggest that exogenous H_2_S, including that derived from the gut microbiota, may serve as a promising therapeutic target for liver fibrosis, in which antioxidative effects appear to play a central and repeatedly observed role.

However, the effects of H_2_S are not uniformly beneficial across all hepatic cell types. In HSCs, exogenous H_2_S has been shown to promote cell proliferation and upregulate fibrogenic gene expression in both primary rat HSCs and bile duct-ligated rat models.^157^ This discrepancy may be explained by differences in experimental models, as well as the route and dosage of H_2_S administration, and indicates the cell-type specific effects of H_2_S within the hepatic microenvironment.

The role of H_2_S in HCC remains controversial, with studies reporting both tumour-promoting and tumour-suppressive effects. On one hand, H_2_S has been shown to promote proliferation, inhibit apoptosis, and enhance angiogenesis and migration of PLC/PRF/5 hepatoma cells via activation of the NF-κB or signal transducer and activator of transcription 3 (STAT3)-cyclooxygenase−2 (COX−2) signalling pathways.^162^^,^^201^ Furthermore, H_2_S supplementation facilitated the sulfhydration of the p65 subunit of NF-κB, thereby increasing its transcriptional activity at anti-apoptotic gene promoters in HEK293 cells.^163^ In contrast, other studies have demonstrated that H_2_S can induce autophagy and apoptosis by inhibiting the phosphatidylinositol 3-kinase (PI3K)/protein kinase-B (AKT)/mTOR signalling pathway.^164^ Additionally, H_2_S attenuates STAT3 activation, thereby contributing to reduced cell proliferation and tumour growth.^165^ These contradictory results may be attributed to differences in the administered dose of H_2_S, as well as the varying sensitivity of different cellular models to its concentration. As evidenced by prior studies, H_2_S appears to exert a dose-dependent biphasic effect in HCC. Low concentrations (10−100 μM) of NaHS (an H_2_S donor) stimulate tumour-promoting behaviours such as proliferation, migration, and angiogenesis, while higher concentrations (800−1000 μM) exert inhibitory effects on tumour growth. This dual action may be mediated through the epidermal growth factor receptor (EGFR)/extracellular signal-regulated protein kinases (ERK)/matrix metalloproteinase 2 (MMP−2) and phosphatase and tensin homologue deleted on chromosome ten (PTEN)/AKT pathways.^161^ Collectively, these studies highlight the complexity of H_2_S -mediated signalling in HCC and underscore its dose-dependent effects.

MT, another sulphur-containing compound, is highly neurotoxic and can induce reversible coma even at low concentrations.^202^ Early clinical studies have suggested a potential association between elevated circulating levels of MT and the occurrence of HE. In healthy individuals, MT concentration in the bloodstream is extremely low, approximately 400 pmol/mL. In patients with liver cirrhosis, the levels increase significantly, even in those without overt HE, and can reach around 1000 pmol/mL in patients with overt HE. Notably, MT levels increased further during HE episodes and exhibited dynamic changes that closely paralleled alterations in mental status. This correlation was stronger than that observed with ammonia levels,^83^ suggesting that MT may contribute to the pathogenesis or progression of HE and could serve as a more sensitive biomarker for neurological deterioration in patients with cirrhosis. However, this interpretation remains contentious. A subsequent study found that although MT was elevated in patients with HE, its increase was the smallest among methionine and its related metabolites and showed no significant variation across different HE severity grades. In addition, the elevated MT levels may partly reflect impaired renal function,^84^ raising doubts about its direct pathological role in HE. Research on MT in HE has remained limited in recent years.

## 4. Translational implications of targeting amino acid metabolism

### 4.1. Biomarker potential of amino acid metabolism

Alterations in amino acid metabolism have been observed across various pathological conditions, providing new opportunities for non-invasive risk assessment and early diagnosis. Accumulating clinical evidence has demonstrated significant associations between circulating amino acid profiles and liver-related diseases. Newgard et al. identified a positive association between elevated plasma levels of BCAAs and IR.^203^ Supporting this observation, subsequent findings from a study in overweight and obese individuals showed that circulating BCAA concentrations were independently linked to hepatic fat accumulation and IR.^204^ Moreover, a large-scale prospective study demonstrated that five amino acids, including both BCAAs (isoleucine, leucine, valine) and AAAs (tyrosine, phenylalanine), were significantly associated with an increased risk of developing T2D. Notably, a model incorporating isoleucine, tyrosine, and phenylalanine significantly improved risk prediction, with individuals in the highest quartile of the amino acid score exhibiting a five- to sevenfold higher risk of developing diabetes compared to those in the lowest quartile, highlighting the potential value of amino acid profiling in early risk assessment.^205^

An untargeted metabolomic analysis of fasting plasma samples from histologically confirmed patients with MASLD (including those with steatosis and MASH) and matched healthy controls revealed that individuals with MASH had significantly elevated circulating levels of phenylalanine, BCAAs (leucine, isoleucine, valine), glutamate, aspartate, and tyrosine. By contrast, patients with simple steatosis showed significant increases in fewer amino acids, including glutamate, lysine, tyrosine, and isoleucine. These metabolomic signatures were insufficient to distinguish hepatic steatosis from steatohepatitis.^206^ A metabolomic analysis of liver biopsy samples from patients with MASLD further confirmed elevated levels of BCAAs in individuals with MASH, aligning with findings from plasma-based profiling. Moreover, hepatic concentrations of BCAAs, tyrosine, and phenylalanine were significantly elevated during the transition from steatosis to MASH.^207^ However, as liver disease progresses, BCAA levels exhibit an opposite trend. In a study of 101 patients with chronic hepatitis and 20 with liver cirrhosis, both plasma BCAA concentrations and the BCAA-to-tyrosine ratio (BTR) progressively declined with increasing fibrosis severity, and were significantly lower in patients with cirrhosis. In contrast, tyrosine levels were elevated even at early stages and continued to rise as the disease advanced.^208^ Consistently, another study involving 137 biopsy-confirmed patients with MASLD also demonstrated that BCAA and BTR levels decreased with fibrosis progression, whereas tyrosine levels positively correlated with both fibrosis stage and IR.^209^ Collectively, these findings underscore a characteristic amino acid imbalance pattern in advanced liver disease, marked by a reduced BTR. This shift is thought to result from impaired hepatic catabolism of AAAs and enhanced peripheral utilisation of BCAAs for energy production and ammonia detoxification,^210^ with important implications for disease staging, nutritional assessment, and metabolic support strategies. Indeed, the BTR has been established as a prognostic factor for early-stage HCC and the most reliable predictor of intrahepatic recurrence.^211^ Moreover, BTR is closely associated with HE and the prognosis of these patients.^212^

Zhang et al. developed a noninvasive diagnostic index for liver fibrosis based on plasma amino acid profiles in patients with chronic hepatitis C. By analysing amino acid concentrations in 53 biopsy-confirmed cases, they derived an optimal index expressed as (Phenylalanine/Valine) + (Threonine + Methionine + Ornithine)/(Proline + Glycine). This composite “amino index” demonstrated strong diagnostic performance, with area under the receiver operating characteristic curve (AUC) values of 0.92 for advanced fibrosis (F3-F4) and 0.99 for cirrhosis. Both sensitivity and specificity exceeded 88% at optimal cut-off values.^213^ A recent large-scale serum metabolomics study involving two independent cohorts of patients with chronic hepatitis B further demonstrated the potential of metabolic profiling for noninvasive fibrosis staging. Using data from 504 HBV-infected patients with liver fibrosis and 502 healthy controls, a panel of four metabolites comprising taurocholate, tyrosine, valine, and linoelaidic acid was identified, and three random forest models were constructed to stratify disease presence and severity. These models exhibited excellent classification performance, achieving AUCs of 0.997 for distinguishing chronic liver disease from healthy controls, 0.941 for differentiating cirrhosis from fibrosis, and 0.918 for staging early versus advanced fibrosis. Validation in an independent cohort of 300 patients with HBV-related chronic liver disease and 90 healthy individuals confirmed the robustness of the models and their superiority over conventional indices, including the aspartate aminotransferase to alanine aminotransferase (AST/ALT) ratio, the AST to Platelet Ratio Index (APRI), and FIB−4 (patient age, AST, ALT, and platelet).^214^ These findings underscore the diagnostic potential of targeted amino acid profiles, either individually or in combination with other metabolite panels, for the noninvasive assessment of liver fibrosis. Future studies should aim to extend these results to liver diseases of diverse etiologies and incorporate longitudinal designs to validate the predictive utility and clinical applicability of metabolite-based indices. Such efforts will be instrumental in facilitating the development of simplified and lower-cost diagnostic tools for routine clinical practice.

Notably, numerous microbial amino acid metabolites have been found to be altered in liver-related diseases (Table 1), with growing mechanistic and preclinical evidence supporting their pathogenic or protective roles (Table 2). Despite this strong biological foundation, large-scale, population-based studies assessing their value as early diagnostic or prognostic biomarkers remain limited. Nonetheless, these mechanistic insights are increasingly driving translational research aimed at developing clinically applicable, noninvasive biomarker strategies. In our recent longitudinal cohort study of patients undergoing transjugular intrahepatic portosystemic shunt (TIPS), elevated baseline serum levels of PEA were independently associated with a sevenfold increased risk of developing HE within three months after the TIPS procedures. These findings suggest that PEA may represent a clinically relevant early warning biomarker for post-TIPS HE.^59^ In addition, disruptions in aromatic and branched-chain amino acid metabolism have been implicated in hepatic steatosis among obese individuals, and PAA was identified as the metabolite most strongly associated with steatosis, highlighting its potential as a biomarker in obesity-related hepatic steatosis.^74^ Collectively, these observations emphasise the emerging clinical relevance of microbial amino acid metabolites within the gut-liver axis. Their potential role in disease prediction underscores warrants further investigation and validation in large-scaler, well-characterised human cohorts to substantiate their robustness and translational value. In parallel, standardised detection technologies and analytical methodologies are needed to ensure consistency, comparability, and clinical applicability across studies.

### 4.2. Therapeutic strategies targeting amino acid metabolism

Emerging discoveries have highlighted amino acid metabolism as a critical axis linking nutrition, gut microbiota, and host pathophysiology. Although a wide range of microbial amino acid metabolites have been mechanistically implicated in the development and progression of liver diseases, clinical interventions directly targeting these metabolites remain largely unexplored. A growing body of preclinical and clinical evidence supports the feasibility of targeting amino acids, either through dietary restriction or supplementation, as a strategy to prevent or treat metabolic and liver-related diseases.

In recent years, multiple clinical studies have explored the impact of modifying dietary protein quantity and composition on the prognosis of liver-related diseases. In patients with MASLD, high-protein diets have shown greater effects in reducing hepatic steatosis compared to low-protein diets. In a randomised controlled trial, 19 patients with morbid obesity received isocaloric low-energy diets with either 10% or 30% of energy from protein. Intrahepatic lipid levels decreased significantly only in the high-protein group, despite similar weight loss. The high-protein diet also reduced hepatic expression of lipid uptake and synthesis genes and attenuated inflammatory signalling. These findings suggest that its benefits were mediated by suppression of hepatic lipid accumulation.^215^ Another multicenter RCT involving 226 overweight or obese patients with MASLD further confirmed these findings. The intervention group followed a low carbohydrate, high protein calorie-restricted diet combined with exercise and intensive counselling from a dietitian. This group exhibited significantly greater reductions in liver fat attenuation parameter, body mass index, and multiple metabolic indexes, including ALT, AST, gamma-glutamyl transferase (GGT), triglycerides, and fasting glucose.^216^ Emerging evidence also suggest that high protein intake may affect host metabolism via the gut microbiota. An intervention study in healthy individuals demonstrated that a high-protein diet enriched microbial species capable of producing secondary bile acids, which was associated with elevated levels of secondary bile acids and glucagon, enhanced amino acid catabolism, reduced visceral adiposity, and lower total cholesterol.^217^ However, in contrast to these findings, protein restriction has shown metabolic benefits in various experimental models of metabolic disease. Protein-restricted diets have been reported to improve metabolic function, reduce the risk of chronic diseases, and promote healthy aging.^218-220^ These contrasting findings suggest that the metabolic effects of dietary protein modulation may depend on factors such as baseline nutritional status, disease stage, and protein source, highlighting the need for personalised dietary strategies in liver disease management.

An important limitation of protein-based interventions is the lack of precision, as some amino acids have been shown to correlate positively with disease risk, while others in the same food source exert protective effects.^221^ This highlights the need for more targeted strategies focusing on specific amino acid types for effective disease management. Among these, modulation of BCAA intake has drawn considerable attention due to its multifaceted effects on metabolic health and liver disease. However, clinical evidence regarding BCAA modulation has shown conflicting outcomes in different stages of liver disease. In MASLD, circulating levels of BCAAs are frequently elevated. Emerging evidence suggests that dietary restriction of BCAAs may confer metabolic benefits, including improved insulin sensitivity, enhanced fatty acid oxidation, and increased energy expenditure.^222-224^ Consistently, a clinical study demonstrated that short term dietary restriction of BCAAs reduced postprandial insulin secretion, improved white adipose tissue metabolism, and modulated gut microbiota composition.^224^ However, comprehensive clinical trial evidence supporting the therapeutic benefits of BCAA restriction in liver diseases remains limited. Conversely, in advanced liver diseases such as liver cirrhosis and HCC, BCAA supplementation is recommended as an effective intervention by multiple clinical guidelines.^225^^,^^226^ A recent systematic review and meta-analysis, comprising 28 studies, compared oral BCAA supplementation to control treatments in patients with liver cirrhosis. The results indicated that oral BCAA supplementation significantly reduced the incidence of HE and other liver-related complications, such as decompensation events. Notably, in a subgroup of patients undergoing surgical resection for HCC, BCAA supplementation further decreased the recurrence of HE and related liver complications.^227^ Although these findings support the role of BCAAs as an adjunct nutritional therapy in liver cirrhosis to reduce complications, an improvement in overall survival has not yet been clearly demonstrated. Additionally, a Cochrane review highlighted differential outcomes based on the route of administration, finding oral BCAA supplementation beneficial for HE, whereas intravenous BCAA did not yield similar benefits.^228^ Given the critical role of gut microbiota as a pathogenic factor in HE, the differential efficacy of oral versus intravenous BCAAs suggests that gut microbiota may mediate some beneficial effects of oral BCAA supplementation, a hypothesis that warrants further investigation.

Another commonly used class of amino acids in dietary interventions is the SAAs, including cysteine and methionine. Modulating SAA intake has been shown to influence diverse diseases. For instance, a randomised controlled trial found that restricting dietary SAAs may exert beneficial effects on systemic metabolic markers, including fibroblast growth factor 21 (FGF21).^229^ Additionally, SAA restriction was shown to protect against Desulfovibrio-induced liver injury by reinforcing hepatic antioxidant and detoxification capacity and restoring bile acid homoeostasis,^230^ suggesting a key role in gut-liver axis. When focusing on individual SAAs, cysteine restriction has been reported to enhance serine biosynthesis and attenuate hepatic triglyceride accumulation, thereby improving lipid metabolism in the liver.^231^ Similarly, recent research has demonstrated that cysteine deficiency triggers systemic metabolic reprogramming, leading to rapid fat mobilisation and consequent weight loss.^232^ This beneficial effect was further supported by an intervention study in obese individuals, where dietary restriction of SAA led to greater weight loss, reduced leptin levels, and increased ketone body production compared to controls. In contrast to restriction, supplementation of SAAs has also shown potential benefits. High-SAA diet resulted in post-translationally modified gut microbial tryptophanase activity, thereby reducing the production of uraemic toxins and attenuating chronic kidney disease progression in mice.^233^ This further underscores the critical role of the gut microbiota in mediating the effects of dietary amino acids on host health.

Engineered microbes represent one of the pivotal strategies for modulating gut microbiota-derived metabolites. Using synthetic biology, bacteria can be designed to produce or consume specific metabolites that precisely modulate specific host metabolic pathways, thereby intervening in disease progression. For instance, one study engineered the probiotic *Escherichia coli* Nissle 1917 (EcN) to efficiently produce AHR ligands, such as IAA and ILA. In an ethanol-induced liver injury model, these metabolites acted on intestinal IL−22-producing cells to activate AhR signalling and enhance IL−22 production, thereby limiting bacterial translocation to the liver and significantly ameliorated hepatic damage.^234^ In another study, an ILA-producing EcN strain also demonstrated beneficial effects in a DSS-induced colitis model.^235^^,^^236^ Additionally, ammonia, a toxic byproduct of intestinal amino acid metabolism, has been targeted by engineering the EcN strain SYNB1020 to convert it to arginine. This strain significantly reduced hyperammonemia in a mouse model of liver injury. Furthermore, a phase 1 clinical study confirmed its safety and successful colonisation, supporting its potential for further clinical development.^237^ These findings collectively highlight the promising application of engineered microbes in therapies that target amino acid metabolism.

## 5. Conclusions and future perspectives

Microbial amino acid metabolism has emerged as a critical component of gut-liver communication, generating a wide array of bioactive metabolites that influence host physiology. Here, we summarise current knowledge regarding its roles in liver diseases, identify the key challenges, and outline prospective directions for future studies.

In basic research, future should address three fundamental questions: 1) What is the microbial contribution to amino acid metabolism in vivo? Many amino acid metabolites are generated through overlapping host and microbial pathways. Distinguishing microbial-derived from host-derived metabolites is essential for interpreting their functional roles. 2) Which microbially derived metabolites are truly relevant to liver disease? For those metabolites primarily produced by microbes, it is crucial to determine whether they are merely associated with liver pathology or actively involved in disease processes. This distinction determines whether a metabolite is better suited as a diagnostic marker, prognostic indicator, or therapeutic target. 3) What are the underlying mechanisms linking these metabolites to liver disease? When causal relationships are established, the next step is to uncover the molecular and cellular mechanisms through which these metabolites act, such as their effects on inflammation, metabolism, fibrosis, or immune signalling. Addressing these questions will require integrated strategies, including isotope tracing, gnotobiotic models, organoid systems, and multi-omics approaches to attribute metabolite origins and functions with precision.

Translationally, microbial amino acid metabolites show strong promise as non-invasive biomarkers and therapeutic targets. Early clinical studies suggest associations between metabolite profiles and disease stages, and emerging interventions, such as amino acid dietary modulation, engineered probiotics, and metabolite supplementation, have shown encouraging results in preclinical models. However, most findings remain preliminary. Moving toward clinical application will require: 1) Large-scale, diverse, and longitudinal human cohorts to validate diagnostic and prognostic utility. 2) Standardised, low-cost detection technologies for reliable metabolite quantification. 3) Stratified clinical trials that account for host genetics, microbiota composition, and dietary habits. With deeper mechanistic insights and robust validation, microbial amino acid metabolism may become a cornerstone of precision diagnostics and targeted interventions in liver disease management.
